# A PCR-based diagnostic testing strategy to identify carbapenemase-producing Enterobacteriaceae carriers upon admission to UK hospitals: early economic modelling to assess costs and consequences

**DOI:** 10.1186/s41512-019-0053-x

**Published:** 2019-04-18

**Authors:** Eoin Moloney, Kai Wai Lee, Dawn Craig, A. Joy Allen, Sara Graziadio, Michael Power, Carolyn Steeds

**Affiliations:** 1 Health Economics Group, Institute of Health & Society, Newcastle University, Baddiley-Clark Building, Newcastle, NE2 4AX UK; 2 NIHR Newcastle In Vitro Diagnostics Co-operative, Newcastle University, Newcastle, UK; 3 NIHR Newcastle In Vitro Diagnostics Co-operative, Newcastle upon Tyne NHS Foundation Trust, Newcastle, UK; 4 Valid Insight, London, UK

**Keywords:** Carbapenemase-producing Enterobacteriaceae, Economic analysis, Cost-consequences, Cost implications, Polymerase chain reaction, Culture, Diagnostic accuracy, Test accuracy

## Abstract

**Background:**

Carbapenemase-producing Enterobacteriaceae (CPE), bacteria which are resistant to the carbapenem class of antibiotics, present an urgent public health risk. The objective of this study was to assess the potential costs and consequences of implementing a testing strategy involving a polymerase chain reaction (PCR)-based diagnostic test for CPE amongst high risk patients upon admission to UK hospitals, to replace the current culture-based testing strategy.

**Methods:**

A decision-analytic model was developed to estimate the expected medical care costs associated with a PCR testing strategy for CPE compared with the current culture testing strategy, and to consider the consequences, in terms of the diagnostic accuracy and associated cost implications, of each approach. The modelled population were patients admitted to hospital at high risk of colonisation with CPE, with model pathways for current practice based on those described in the Public Health England (PHE) toolkit for CPE testing. Costs were estimated from a UK National Health Service (NHS) perspective, with outcomes presented in terms of percentage of samples identified as true positive, false positive, true negative and false negative following each method of testing.

**Results:**

Results indicated that the PCR testing strategy led to an estimated cost saving of £462 per patient for a 5-day hospital stay. For all sensitivity analyses conducted, PCR testing resulted in an expected cost saving. Potential cost savings approached £850 per patient for the sensitivity analysis assuming a 15-day hospital stay, indicating that PCR testing results in greater cost savings as length of stay increases. Fewer false positive, and more true negative, cases were identified with the PCR testing strategy in all analyses conducted.

**Conclusions:**

This economic analysis gives an insight into the potential cost savings that could be made by the UK NHS through the introduction of a PCR-based diagnostic testing strategy to replace current recommended culture-based methods for the detection of CPE. Savings are due primarily to a faster time to result with PCR, meaning that CPE-free patients are not isolated unnecessarily. Therefore, a PCR-based diagnostic may aid appropriate use of isolation resource.

## Background

Carbapenemase-producing Enterobacteriaceae (CPE) present a threat to healthcare and are an urgent public health risk [1]. These bacteria are resistant to the carbapenem class of antibiotics, often considered the “last resort” in the treatment of many bacterial infections. CPEs restrict treatment options for patients and are associated with increased morbidity and mortality [2]. They are readily transmissible in health care settings, and countries such as Greece and Italy are already considered endemic for some strains of CPE [2]. Patients may be colonised or infected with CPE. With colonisation, these bacteria normally live harmlessly on the skin or in the bowel, but, if they enter into other areas such as the bladder or bloodstream, they can cause disease. However, even when asymptomatic, colonised patients can transmit CPE, which means that strict precautions need to be taken to avoid onward spread of these potentially harmful organisms by patients suspected, or at risk, of colonisation or infection. Recent research has estimated the cost of an outbreak of CPE in a London hospital group at over £1 million over 10 months [3], which makes a strong economic case for the early identification of patients colonised with CPE, and associated infection control procedures.

There is currently no gold standard screening method for CPE [4]. However, current Public Health England (PHE) guidelines recommend that high risk patients (those previously admitted to hospital within specific geographical areas both inside and outside the UK, or who have previously been identified as CPE-positive) should be immediately isolated from the general patient population upon hospital admission [5]. It is advised that these patients should be screened for CPE, typically via culture on a commercially-produced chromogenic agar designed for the identification of CPE. Isolation is recommended until three consecutive negative results are obtained: stool samples taken via rectal swabbing on admission day (0), and days 2 and 4 after admission. These tests can take 48 h to produce a negative result meaning that, if guidelines were followed, patients could be isolated for almost a week upon admission, in many cases unnecessarily given the low prevalence of CPE currently within the UK. Rapid polymerase chain reaction (PCR) tests for the detection of the major carbapenemase gene families (*Klebsiella pneumoniae* carbapenemase (KPC), New Delhi metallo-beta-lactamase (NDM), Imipenemase (IMP), Verona imipenemase (VIM) and OXA-48-like have the potential to quickly stratify patients according to the presence or absence of any of the 5 key carbapenemase genes from a single sample, with results produced in a few hours rather than days. Such tests facilitate early diagnosis and appropriate patient management, allowing prompt deployment of infection prevention and control measures only when there is a real need.

Early health economic models are tools which can be used to predict the economic viability of new medical technologies or health care interventions [6]. They can be used to explore possible outcomes and areas of uncertainty and inform the design of future research, as well as directly inform the design of the technology itself. Through an early decision-analytic modelling exercise, this study estimates the costs and consequences of replacing the current culture-based diagnostic testing strategy with a PCR-based diagnostic strategy to test for CPE in UK hospitals, and therefore identify the key health and cost outcomes likely to improve if this technology were to be adopted.

## Methods

### Model overview

**Fig. 1:**
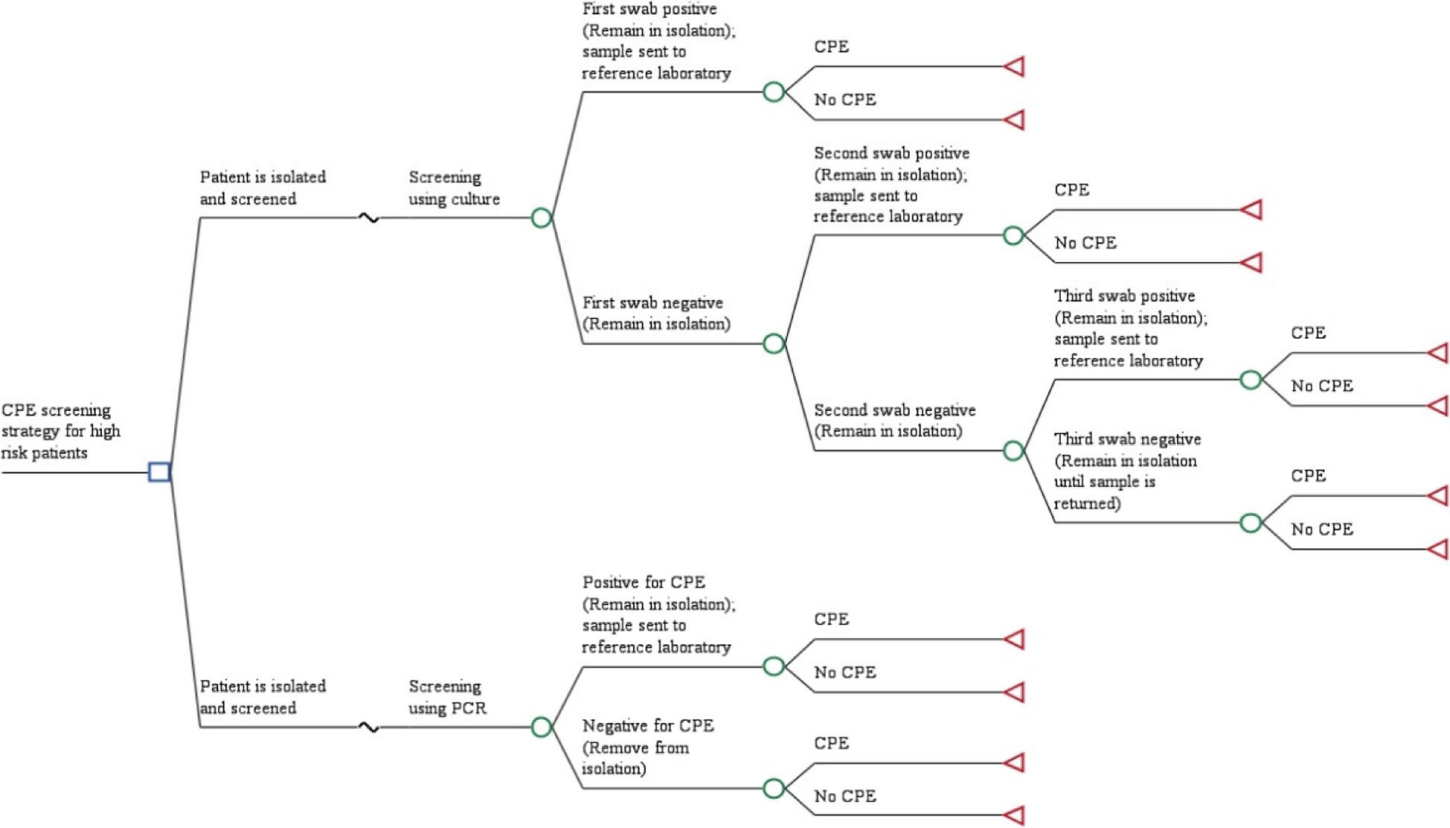
Decision tree to assess costs and consequences of PCR testing for CPE compared with culture testing for CPE

A decision tree was built in TreeAge Pro® 2016 [7] to estimate the expected cost of CPE screening with PCR compared with standard culture testing, and to consider the likely cost implications of an incorrect patient diagnosis based on the diagnostic accuracies of the two testing strategies. The population was patients admitted to hospital who were considered to be at high risk of colonisation with CPE according to criteria defined in the Public Health England (PHE) acute trust toolkit for early detection, management and control of CPE [5]. The economic model was designed to assess costs associated with testing and subsequent patient infection control management over a 5-day period. The time horizon of the model was based on the mean length of stay in hospital for patients across all specialities, informed by Hospital Episode Statistics (HES) 2016/17 [8]. The test performance estimates for the culture and PCR tests being evaluated are not based on data for any one specific test, with estimates instead derived through a systematic review and meta-analysis of relevant tests, and cost and resource use data derived through the literature and expert clinical input. The model structure was based on the patient pathways described in the PHE toolkit [5] and is shown in Fig. 1.

All patients in the model begin at a point where they are defined as high risk and are immediately isolated. These patients are subject to strict standard precautions to prevent possible spread and undergo one of the two alternative screening strategies for the detection of the presence of CPE:

### Screening using culture (current practice)

With management based on culture results, the patient is immediately placed in side room isolation and rectal swabs are taken on days 0, 2 and 4 with negative results returned after 48 h. Should any of the swab samples test positive for CPE, the patient is required to remain in the isolation room for the duration of their hospital stay. This positive sample would then undergo species identification by Matrix-assisted laser desorption/ionisation – time-of-flight mass spectrometry (MALDI-TOF MS) as a confirmatory test. MALDI-TOF MS is a rapid, accurate and cost-effective method of microbial characterisation and identification [9]. Carba-NP phenotypic testing, and antibiotic susceptibility testing at the hospital laboratory would then be conducted. It would then be sent to the antimicrobial resistance and healthcare associated infections (AMRHAI) reference unit to identify the carbapenemase gene for epidemiological surveillance purposes. These follow-up confirmatory tests are included in the model for cost purposes only, with modelled diagnostic accuracy based on the culture or PCR test alone. Per PHE guidelines, three consecutive negative culture test results are required before a patient is released from isolation. Therefore, the patient remains in isolation until discharge on the fifth day, before the results from swabs collected on day 4 are returned.

### Screening using PCR

With PCR testing, the patient is immediately placed in an isolation room and swabbed on admission. Results are returned on the same day (day 0). Where the PCR test returns negative, the patient is placed in a general ward until discharge on the fifth day. For these patients, cost of isolation is only included for the first day as it is assumed that results are available on the day of admission. Where the PCR test returns positive, the patient remains in the isolation room. Any positive sample would then undergo culture testing, species identification by MALDI-TOF MS, antibiotic susceptibility testing at the hospital laboratory and would then be sent to the AMRHAI reference unit for epidemiological surveillance purposes. The patient would remain in isolation until discharge on the fifth day.

All model assumptions regarding subsequent testing of samples and patient infection control management were based on expert clinical input and the pathways described in the PHE toolkit [5], respectively.

### Estimation of model parameters

#### Resource use and costs

Quantifying the resources required for each screening strategy was informed through a combination of evidence from published literature and expert clinical input. A microbiology laboratory manager at the Newcastle upon Tyne Hospitals NHS Foundation Trust provided list prices for culture testing, PCR testing, antibiotic susceptibility testing, and delivery of swab samples to the AMRHAI reference laboratory. These included associated test/consumable and labour costs, based on expert clinical advice provided by the aforementioned laboratory manager. All of the costs provided by the laboratory manager reflect the costs incurred at that specific laboratory, but these costs may vary across laboratories.

The cost of performing a Carba NP test was sourced from a study by Yusuf et al. (2014) [10]. The cost used was based on the cost of the commercial Rapid CARB Screen kit included in this study. As costs in this study were expressed in €, an exchange rate of £1: €1.25 (2014 exchange rate; price year of costs included in the study) was applied to convert costs to pound sterling. This cost included test costs only, and labour costs were added to this based on expert clinical advice from the microbiology laboratory manager. The cost of performing species identification by MALD-TOF MS was sourced from a study by Tran et al. (2015) [11]. As costs in this study were expressed in $, an exchange rate of £1: $1.70 (2014 exchange rate; price year of costs included in the study) was applied to convert costs to pound sterling. This cost included associated test, labour, and maintenance costs.

The cost of consumables for patient swabbing, the additional cost of an isolation room and the cost of contact precautions were taken from a *Department of Health report for methicillin-resistant Staphylococcus aureus* (MRSA) [12], which in turn sourced costs from the *Scottish MRSA Screening Pathfinder Programme* [13]. It was assumed that these costs, although related to MRSA, would be broadly similar if applied to CPE. Costs of hospital stay were taken from *NHS reference costs 2016/17* [14]. Where costs were sourced from work conducted prior to 2017, costs were inflated to 2017 prices according to the hospital and community health services (HCHS) index listed in the *Unit costs of health and social care 2017* [15]. Resource use items and associated costs expressed in 2017 UK prices are presented in Table 1.

**Table 1:** Resource use items and costs

Cost item	Unit cost (£)	Source
Culture testing (staff & test costs)	8.79	Expert clinical input^a^
PCR testing (including DNA extraction) (staff & test costs)	39.59	Expert clinical input^a^
Consumables for swabbing	3.62	MRSA Pathfinder (Scotland) 2008/2009 [13]
Presumptive confirmation by phenotypic test (Carba NP) (staff & test costs)	2.78	Yusuf, E. et al. (2014) [10]
Species identification by MALDI-TOF (staff & test costs)	1.92	Tran, A. et al. (2015) [11]
Referral to AMRHAI reference laboratory (staff & consumable costs)	0.65	Expert clinical input^a^
Antibiotic susceptibility testing (staff & consumable costs)	3.99	Expert clinical input^a^
Non-elective bed day	303.74	NHS reference costs 2016/2017 [14]
Isolation room bed day^b^	100.12	MRSA Pathfinder (Scotland) 2008/2009 [13]
Contact precaution per day	22.12	MRSA Pathfinder (Scotland) 2008/2009 [13]

^a^Cost based on information provided by a microbiology laboratory manager at the Newcastle upon Tyne Hospitals NHS Foundation Trust. Costs are likely to be representative of other tertiary hospitals, however they may vary

^b^Isolation room bed day costs incurred in addition to non-elective bed day costs

### Sensitivities and specificities of the culture and PCR screening strategies

To inform test performance estimates for the economic modelling base-case analysis, systematic searches were performed in MedLine and EMBASE for publications of commercial culture and PCR tests used for CPE detection, including conference abstracts. These searches were performed in June, 2017. Studies were included only if they tested rectal swabs, perirectal swabs or stool samples, and if they tested for CPE. Two reviewers independently screened for studies which met the inclusion criteria. Eight full papers [16–23] were included for commercial culture tests and two full papers and two conference abstracts [4, 24–26] were included for commercial PCR tests. The selected papers were quality assessed using the Quality Assessment of Diagnostic Accuracy Studies (QUADAS-2) tool [27]. As there is no gold standard test for screening for CPE, the reference standard varied across the identified studies. For the identified commercial culture test studies, the risk of bias with regard the reference standards was low as they were chosen to be likely to correctly diagnose CPE. Similarly, for the identified commercial PCR test studies, the risk of bias was low as they used alternative PCR tests or standard workflow based on culture and phenotypical identification of the colonies as the reference standard. In addition, for both groups of studies, authors ensured that the index test results would not affect the interpretation of the reference standard results.

Separate meta-analyses were subsequently performed for the culture and PCR tests identified through the systematic search. Meta-analyses of the test accuracy results were fitted according to the bivariate random effects model in STATA [28] using the “metandi” command and the “gllamm” option to estimate the mean sensitivities and specificities for the culture and PCR studies. The meta-analysis results, with 95% confidence intervals also presented, were: culture; sensitivity = 0.834 (CI 0.704–0.914), specificity = 0.933 (CI 0.843–0.973), and PCR; sensitivity = 0.960 (CI 0.759–0.994), specificity = 0.966 (0.898–0.989).

Test performance estimates for the Carbaplex® assay (the development and validation of which was being conducted alongside this work) were also derived from performance evaluations carried out on prospective samples (sensitivity = 1.000 (CI 0.753–1.000), specificity = 0.982 (CI 0.976–0.987)) [29]. These values would be used to inform a sensitivity analysis, described later.

### Prevalence of CPE

A recent, reliable, estimate of the prevalence of CPE across the UK as a whole was unavailable and so, an estimate of prevalence in the North West of England was used as a proxy. The estimate for prevalence included in the base-case economic model was based on a report compiled by PHE in July 2016 with the aim of supporting trusts in implementing the CPE toolkit and improving understanding of the epidemiology of Carbapenem-resistant Enterobacteriaceae (CRE) in the Greater Manchester area [30]. The report detailed the testing of a number of secondary care patients across the North West of England for the presence of CRE, from a variety of specimen types. A prevalence of 0.6% was reported.

### Assessment of costs and diagnostic accuracy classifications

The model was run deterministically to obtain the expected values for both strategies. The analysis was designed to generate the incremental cost per patient of introducing a PCR-based diagnostic approach to test for CPE in order to replace current culture-based methods. Costs were estimated from the perspective of the UK National Health Service (NHS), with costs dependent on the probabilities of patients moving through the different branches of the model according to their test results.

Given the sensitivities and specificities of the culture and PCR tests, and the prevalence of CPE in the patient population, for the first test (culture or PCR strategy) we can calculate the diagnostic accuracy classifications for the two testing strategies:

$$ {\displaystyle \begin{array}{c}{\mathrm{TP}}_1={\mathrm{Prevalence}}^{\ast }\ \mathrm{Sensitivity}\\ {}{\mathrm{FN}}_1={\mathrm{Prevalence}}^{\ast }\ \left(1-\mathrm{Sensitivity}\right)\\ {}{\mathrm{TN}}_1={\left(1\hbox{-} \mathrm{Prevalence}\right)}^{\ast }\ \mathrm{Specificity}\\ {}{\mathrm{FP}}_1={\left(1\hbox{-} \mathrm{Prevalence}\right)}^{\ast }\ \left(1-\mathrm{Specificity}\right)\end{array}} $$

With PCR testing, there is only one test. For the second and third culture tests (*n* = 2, 3), we calculate:

$$ {\displaystyle \begin{array}{l}\begin{array}{c}{\mathrm{Prevalence}}_{\mathrm{n}}={\mathrm{FN}}_{\left(\mathrm{n}-1\right)}/\left({\mathrm{FN}}_{\left(\mathrm{n}-1\right)}+{\mathrm{TN}}_{\left(\mathrm{n}-1\right)}\right)\\ {}{\mathrm{TP}}_{\mathrm{n}}={{\mathrm{Prevalence}}_{\mathrm{n}}}^{\ast }\ \mathrm{Sensitivity}\\ {}{\mathrm{FN}}_{\mathrm{n}}={{\mathrm{Prevalence}}_{\mathrm{n}}}^{\ast }\ \left(1-\mathrm{Sensitivity}\right)\end{array}\\ {}\begin{array}{c}{\mathrm{TN}}_{\mathrm{n}}={\left(1\hbox{-} {\mathrm{Prevalence}}_{\mathrm{n}}\right)}^{\ast }\ \mathrm{Specificity}\\ {}{\mathrm{FP}}_{\mathrm{n}}={\left(1\hbox{-} {\mathrm{Prevalence}}_{\mathrm{n}}\right)}^{\ast }\ \left(1-\mathrm{Specificity}\right)\end{array}\end{array}} $$

### Base-case analysis

The base-case analysis included the following parameter values:

- Prevalence of CPE = 0.6%
- Culture sensitivity = 0.834, specificity = 0.933
- PCR sensitivity = 0.960, specificity = 0.966
- Hospital stay = 5 days

### Sensitivity analysis

Sensitivity analyses were performed to determine the impact of changing key parameters on the model results. Therefore, many of the model parameters were subject to one-way sensitivity analysis, using hypothetical increases or decreases, to explore the impact that it would have on the model results.

The following scenarios were explored:

- Based on clinical input, patients may sometimes be removed from isolation after 1 negative culture test due to resource constraints on isolation rooms. Therefore, the number of consecutive negative culture tests required before a patient is removed from isolation was varied.
- The impact of extending length of stay in hospital to 15 days was explored.
- 2015 data from the European Centre for Disease Prevention and Control’s (ECDC) Surveillance Atlas on antimicrobial resistance [31] indicated that the proportion of carbapenem-resistant isolates was as high as 33.5% and 61.9% in Italy and Greece, respectively. Therefore, in order to explore an increased prevalence of CPE scenario, the prevalence parameter was increased to 5% to assess costs and consequences amongst what was considered to be a higher risk population.
- As an alternative to the sensitivity and specificity estimates for PCR used in the base-case analysis (derived from the systematic review and meta-analysis), test performance estimates for the Carbaplex® assay [29] were assigned to PCR to explore the impact that this would have on results.
- The cost of running a PCR test was more than doubled to £80.
- Based on clinical input, the turnaround time for PCR results may be variable and it might be that same-day availability of results is not possible. Therefore, a scenario was explored whereby patients who ultimately receive a negative PCR result are required to remain in isolation for the first two days of their hospital stay

## Results

### Base-case analysis

The base-case economic analysis results shown in Table 2 indicate that the expected cost with PCR testing is £462.13 less per patient than with culture testing; PCR testing is a cost saving strategy. The diagnostic accuracy results indicate that a greater percentage of the patient cohort would be classified as FP after three culture tests than after the PCR test, while fewer patients would be classified as TN following culture testing compared with PCR.

**Table 2:** Base-case analysis expected costs and test performance

Strategy	Expected cost per patient (£)	True positives (%)	False positives (%)	True negatives (%)	False negatives (%)
Culture testing	2166.37	0.6	18.7	80.7	0.0027
PCR testing	1704.24	0.6	3.4	96	0.024
Cost difference with PCR testing (£)	−462.13				

### Sensitivity analyses

#### One negative culture

Results of sensitivity analysis (a), where one negative culture test is assumed sufficient to remove a patient from isolation, indicate that the expected cost with PCR testing is £211.76 less per patient than with culture testing; PCR testing remains cost saving (Table 3). The diagnostic accuracy results indicate that a greater percentage of the cohort would be classified as TP and TN following PCR testing, and a smaller percentage as FP and FN, compared with culture testing.

**Table 3:** Sensitivity analyses expected costs and test performance

Strategy	Expected cost per patient (£)	True positives (%)	False positives (%)	True negatives (%)	False negatives (%)
Sensitivity Analysis (a):					
Culture testing	1916.00	0.5	6.7	92.7	0.0996
PCR testing	1704.24	0.6	3.4	96.0	0.024
Cost difference with PCR testing (£)	−211.76				
Sensitivity Analysis (b):					
Culture testing	5639.07	0.6	18.7	80.7	0.0027
PCR testing	4791.70	0.6	3.4	96.0	0.024
Cost difference with PCR testing (£)	−847.37				
Sensitivity Analysis (c):					
Culture testing	2165.82	5.0	17.8	77.2	0.023
PCR testing	1724.94	4.8	3.2	91.8	0.2
Cost difference with PCR testing (£)	−440.88				
Sensitivity Analysis (d):					
Culture testing	2166.37	0.6	18.7	80.7	0.0027
PCR testing	1696.29	0.6	1.8	97.6	0
Cost difference with PCR testing (£)	−470.08				
Sensitivity Analysis (e):					
Culture testing	2166.37	0.6	18.7	80.7	0.0027
PCR testing	1744.65	0.6	3.4	96	0.024
Cost difference with PCR testing (£)	−421.72				
Sensitivity Analysis (f):					
Culture testing	2166.37	0.6	18.7	80.7	0.0027
PCR testing	1821.65	0.6	3.4	96	0.024
Cost difference with PCR testing (£)	−344.72				

#### 15-day stay in hospital

Results of sensitivity analysis (b), where a 15-day stay in hospital is assumed, indicate that the expected cost with PCR testing is £847.37 less per patient than with culture testing (Table 3). Diagnostic accuracy results do not change from the base-case analysis as they are not impacted by length of stay.

#### High-risk population (5% prevalence)

Results of sensitivity analysis (c), where a 5% prevalence of CPE is assumed, indicate that the expected cost with PCR testing is £440.88 less per patient than with culture testing; PCR testing remains cost saving (Table 3). Diagnostic accuracy results are similar to those seen in the base-case analysis.

#### Carbaplex® sensitivity & specificity (sensitivity = 1.000 (CI 0.753–1.000), specificity = 0.982 (CI 0.976–0.987))

Results of sensitivity analysis (d), where sensitivity and specificity estimates for Carbaplex® [29] were applied to the relative PCR values, indicate that the expected cost with PCR testing is £470.08 less per patient than with culture testing (Table 3).

#### Increased cost of PCR

Results of sensitivity analysis (e), where the cost of running a PCR test was increased to £80, indicate that the expected cost with PCR testing is £421.72 less per patient than with culture testing (Table 3). Diagnostic accuracy results are as seen in the base-case analysis.

#### Longer turnaround time for PCR results

Results of sensitivity analysis (f), where the turnaround time for results from the PCR test are increased and patients who ultimately receive a negative result are required to remain in isolation for the first two days of their hospital stay, indicate that the expected cost with PCR testing is £344.72 less per patient than with culture testing (Table 3). Diagnostic accuracy results are as seen in the base-case analysis.

## Discussion

An economic model was developed to estimate the costs and diagnostic accuracies associated with culture and PCR testing strategies for CPE amongst adults attending the UK NHS who are at a high risk of carrying CPE. Parameter values were varied in sensitivity analysis to determine the impact that this would have on the model outcomes and results.

In the base-case analysis, although the cost of the individual PCR test is more expensive than the cost of a culture test, cost savings are made primarily due to the fact that a PCR test allows for the same-day removal of patients from isolation upon the return of a negative result while, according to PHE guidelines, patients receiving a culture test are required to remain in isolation until three consecutive negative results are obtained. This finding is consistent for all sensitivity analyses presented, even when the number of tests for each screening strategy is identical. In this analysis, although testing sequences are the same for the two strategies, culture is still more expensive due to the fact that patients remain in isolation for the first two days while awaiting results. Patients receiving a PCR test, on the other hand, can be removed from isolation on the same day as the test is conducted, provided the results are negative, indicating that the rapid turnaround of results drives cost savings. Potential cost savings approach £850 per patient for the sensitivity analysis assuming a 15-day hospital stay. In a further sensitivity analysis assuming that all patients receiving a PCR test must remain in isolation for at least two days (increased to full duration of hospital stay for positive test patients), the expected cost of PCR testing is still less than culture testing due to the assumption that repeat testing on negative samples is not required and patients can be removed from isolation as soon as the negative result is received.

In the base-case analysis, the diagnostic accuracy results suggest that a larger percentage of the patient cohort are identified as FP after three consecutive culture tests than after the single PCR test conducted. More incorrect diagnoses are recorded due to the imperfect nature of the test and the low prevalence of CPE. From a hospital perspective, the direct and indirect economic implications of isolating patients that do not need isolation are large. In addition to the direct costs of isolating the patient and enforcing strict standard precautions, there are also the opportunity costs of utilising resources that have alternative uses. Isolation may also have an adverse effect on patients in terms of quality of life and satisfaction [32]. For all analyses conducted, other than when treatment sequences are identical and the sensitivity and specificity values of PCR are varied, a marginally higher percentage (approx. 0.02–0.2%) of patients are identified as FN with PCR testing (based on meta-analysis findings) compared with culture. The risk of FN patients relates to the potential danger of onward transmission and associated economic implications. A full evaluation of these was beyond the scope of this study, however, a recent economic analysis estimated the cost of an outbreak of CPE in a London hospital group to be significant [3].

There are limitations to the analysis, many of which are a consequence of a paucity of published data. The model structure may be viewed as somewhat simplistic. The base-case model only captured costs and outcomes incurred over a 5-day time horizon (average length of stay for patients in hospital across all specialities). No average length of stay data were available for high risk patients; therefore, length of stay parameters included in the model were based on data from a general patient population. However, increased length of stay (15 days) was explored in sensitivity analysis and cost savings associated with PCR increased. There was also a risk of inapplicability of the studies used to estimate the sensitivities and specificities of PCR and culture testing included in the model, as these studies were typically conducted in countries with endemic CPEs and focussed only on one or two specific CPE variants rather than the five that are most common in the UK [33]. There is further uncertainty around the prevalence estimate included in the model as it was sourced from a report which may not be generalizable to the entire UK population, and again this value was varied in sensitivity analysis with PCR still appearing to be the least costly strategy. The model assumes that diagnostic accuracy is dependent on the culture or PCR test alone, with follow-up confirmatory testing included for cost purposes only. This is unlikely to be the case in clinical practice, where decisions are likely to be made on the basis of a combination of tests (particularly in the case of culture). Despite these limitations, a key strength of the economic analysis is that it is, as far as we are aware, the first economic decision-analytic model to consider the relative costs and consequences of PCR and culture testing strategies for CPE in a UK setting. The scarcity of comparable economic modelling studies was highlighted in a search for model-based economic evaluations that was performed to help inform our own model structure. Only two studies [34, 35] involving economic models were identified, and these were both based on the same empirical evidence. The focus of these studies was on active surveillance in Intensive Care Units, and were not considered appropriate in helping to inform our own model structure.

This study suggests that a PCR-based testing strategy for CPE has the potential to be cost saving to the NHS, however further research is required to fully evaluate the level of cost saving that might be achieved through its implementation. In the short term, this would, in part, be driven by the current availability of PCR for CPE testing across UK hospital laboratories. There are no published data to inform current availability, however, based on the expert clinical advice of a microbiology laboratory manager at the Newcastle upon Tyne NHS Hospitals Foundation Trust, it is predicted that the vast majority of UK laboratories have PCR equipment available for the routine testing of bacterial and/or viral pathogens for which culture methods are not appropriate, e.g. chlamydia or influenza., and that these PCR systems could be used for the detection of CPE with the purchase of an appropriate kit. Results of this analysis should be interpreted with caution given the uncertainty in important input parameter values, most notably the test performance estimates and the estimate of CPE prevalence. However, the analysis presented provides a strong platform for discussion and highlights a clear need for further research into potential cost-effectiveness.

## Conclusions

This early economic model gives an insight into the potential cost savings to the NHS by a universal switch to a PCR-based diagnostic testing strategy for the detection of patients carrying a CPE, given current UK screening and patient management guidelines.

## Abbreviations

AMRHAI: Antimicrobial Resistance and Healthcare Associated Infections
CPE: Carbapenemase-producing Enterobacteriaceae
CRE: Carbapenem-resistant Enterobacteriaceae
ECDC: European Centre for Disease Prevention and Control
HCHS: Hospital and Community Health Services
HES: Hospital Episode Statistics
IMP: Imipenemase
KPC: *Klebsiella pneumoniae* Carbapenemase
MALDI-TOF MS: Matrix-Assisted Laser Desorption/Ionisation – Time-of-Flight Mass Spectrometry
MRSA: Methicillin-Resistant *Staphylococcus aureus*
NDM: New Delhi Metallo-Beta-Lactamase
NHS: National Health Service
PCR: Polymerase Chain Reaction
PHE: Public Health England
QUADAS-2: Quality Assessment of Diagnostic Accuracy Studies
VIM: Verona Imipenemase
